# Defective lung function following influenza virus is due to prolonged, reversible hyaluronan synthesis

**DOI:** 10.1016/j.matbio.2018.06.006

**Published:** 2019-07

**Authors:** Thomas J. Bell, Oliver J. Brand, David J. Morgan, Samira Salek-Ardakani, Christopher Jagger, Toshifumi Fujimori, Lauren Cholewa, Viranga Tilakaratna, Jörgen Östling, Matt Thomas, Anthony J. Day, Robert J. Snelgrove, Tracy Hussell

**Affiliations:** a Manchester Collaborative Centre for Inflammation Research, University of Manchester, UK; b Inflammation, Repair and Development, National Heart and Lung Institute, Imperial College London, UK; c Wellcome Trust Centre for Cell-Matrix Research, Division of Cell-Matrix Biology & Regenerative Medicine, School of Biology, Faculty of Biology, Medicine & Health, The University of Manchester, Manchester Academic Health Science Centre, Manchester M13 9PL, UK; d Respiratory, Inflammation & Autoimmunity IMED, AstraZeneca, Gothenburg, Sweden

**Keywords:** HA, hyaluronan, Has2, HA synthase 2, 4MU, 4-methylumbelliferone, HYAL, hyaluronidase, Lung, Matrix, Lung function, Influenza, Inflammation, Fibroblasts

## Abstract

Little is known about the impact of viral infections on lung matrix despite its important contribution to mechanical stability and structural support. The composition of matrix also indirectly controls inflammation by influencing cell adhesion, migration, survival, proliferation and differentiation. Hyaluronan is a significant component of the lung extracellular matrix and production and degradation must be carefully balanced. We have discovered an imbalance in hyaluronan production following resolution of a severe lung influenza virus infection, driven by hyaluronan synthase 2 from epithelial cells, endothelial cells and fibroblasts. Furthermore hyaluronan is complexed with inter-α-inhibitor heavy chains due to elevated TNF-stimulated gene 6 expression and sequesters CD44-expressing macrophages. We show that intranasal administration of exogenous hyaluronidase is sufficient to release inter-α-inhibitor heavy chains, reduce lung hyaluronan content and restore lung function. Hyaluronidase is already used to facilitate dispersion of co-injected materials in the clinic. It is therefore feasible that fibrotic changes following severe lung infection and inflammation could be overcome by targeting abnormal matrix production.

## Highlights

• Influenza causes prolonged changes in hyaluronan due to increased synthase activity
• Influenza induces persistent hyaluronan cross-linking by inter-alpha-inhibitor heavy chains
• Pockets of persistent hyaluronan are associated with CD44-expressing macrophages
• Digestion of hyaluronan with intranasal hyaluronidase restores lung function but upon cessation of treatment post-viral complications return

## Introduction

Extracellular matrix not only provides physical support to tissues but also orchestrates the differentiation, proliferation, migration, positioning and survival of resident and infiltrating immune cells. Organ-specific matrices also provide tissue-specific training of cells that reside within; an interaction that changes profoundly during the tissue destruction and remodelling that occurs in many lung pathologies [1]. Hyaluronan (HA) is a glycosaminoglycan component of the extracellular matrix found at high concentrations in the lung [[2], [3], [4], [5]]. Its synthesis and degradation is mediated by plasma membrane-located HA-synthases (HAS-1-3) [6, 7] that extrude HA from the cell surface [8] and various hyaluronidases [9, 10], respectively. Clearance of HA is a critical requirement in disease resolution. However, in conditions of endoplasmic stress, viral infections, hyperglycaemia and adrenergic receptor stimulation, HA can form cable-like structures that disrupt tissue architecture and are more adhesive to inflammatory cells [11].

Since HA affects water homeostasis, cell-matrix signalling, tissue healing, inflammation, angiogenesis, and cell migration, the balance of HA synthesis to degradation is important to prevent pathological consequences. Accumulation of HA is observed in ovalbumin and house dust mite murine models of allergic airway inflammation [12], bleomycin and ozone induced lung damage [13, 14], LPS and Staphylococcal enterotoxin B induced inflammation [[15], [16], [17]]. In human disease airway hyaluronan is elevated in patients with chronic obstructive pulmonary disease (COPD) and associated with poor lung function, asthma, idiopathic arterial pulmonary hypertension and acute respiratory distress syndrome, amongst others (for a review see [3] and references within).

In the healthy lung, high molecular weight HA is located in peribronchial and perialveolar regions where it provides structural integrity and displays anti-inflammatory properties [18, 19]. During inflammation, however, hyaluronidases and reactive oxygen species (ROS) [18, [20], [21], [22]] induce lower molecular weight HA that is more widely distributed and often observed surrounding alveolar macrophages [23]. Low molecular HA interacts with CD44 and RHAMM [[24], [25], [26]] and is reported to be pro-inflammatory [[27], [28], [29]], however, some of these effects may be due to contaminating endotoxin in the HA preparations used [30]. Furthermore, high molecular weight HA blocks the pro-inflammatory effects of low molecular weight HA in some studies [6, 19]. For a review see [5].

HA can also associate with other proteins that support its deposition [[31], [32], [33], [34]] including the heavy chains from the hyaladherin, inter-α-inhibitor (IαI) in the presence of TNF-stimulated gene-6 (TSG-6) [31, [35], [36], [37]]. TSG-6, originally identified in TNF-stimulated fibroblasts, is absent in most healthy adult tissues, but up-regulated in a variety of cell types during inflammation (for a review see [36, 38]) including fibroblasts [39], epithelial cells [40], vascular smooth muscle [41] and monocytes [42, 43]. There is evidence that TSG-6 is present constitutively in lung BAL fluid and tracheal aspirates, but elevated in asthma and in smokers [44]. TSG-6 is also constitutively expressed in brain, skin and pancreas [[45], [46], [47]]. IαI however, is a serum proteoglycan consisting of three polypeptides, two heavy chains each of ~80 kDa, and a light chain, also known as bikunin of ~25 kDa that confers protease inhibitory properties [44, 48]. IαI is produced by hepatocytes and circulates in the blood stream, leaking into tissues at sites of inflammation; it may also be synthesised locally, e.g. in lung epithelia [49] and the amniotic membrane [50], but definitive data for this are lacking. In the lung, IαI promotes epithelial repair after injury [49]. However, when combined with TSG-6 and HA it facilitates the deposition of a more “pathological matrix”, which binds with altered affinity to CD44 [51, 52] and the lymphatic vessel endothelial hyaluronan receptor-1 (LYVE-1) [53]. Furthermore, in murine allergic asthma, the presence of HC·HA complexes appears to worsen airway hyper-reactivity and eosinophilic inflammation, but not the development of a Th2 immune response [54].

Though extracellular matrix is known to increase during inflammation, little is known about matrix dynamics in inflammatory resolution and beyond. Using a self-resolving influenza infection model we show that hyaluronan persists due to HA-synthase 2 activity in non-immune cells, that macrophages are trapped within this matrix, that HA is significantly modified by addition of HCs from IαI and that the system can be reversed, leading to improved lung function, by administration of haluronidase intransally. However, upon removal of hyaluronidase the system reverts back to its altered state. The signals governing HA-synthase 2 activity are not known. However, hyaluronidase treatment or HA-synthase 2 blockade should be considered to reduce extracellular matrix and release the body of macrophages trapped in it.

## Results

### Hyaluronan accumulates in the airways during influenza infection

**Fig. 1:**
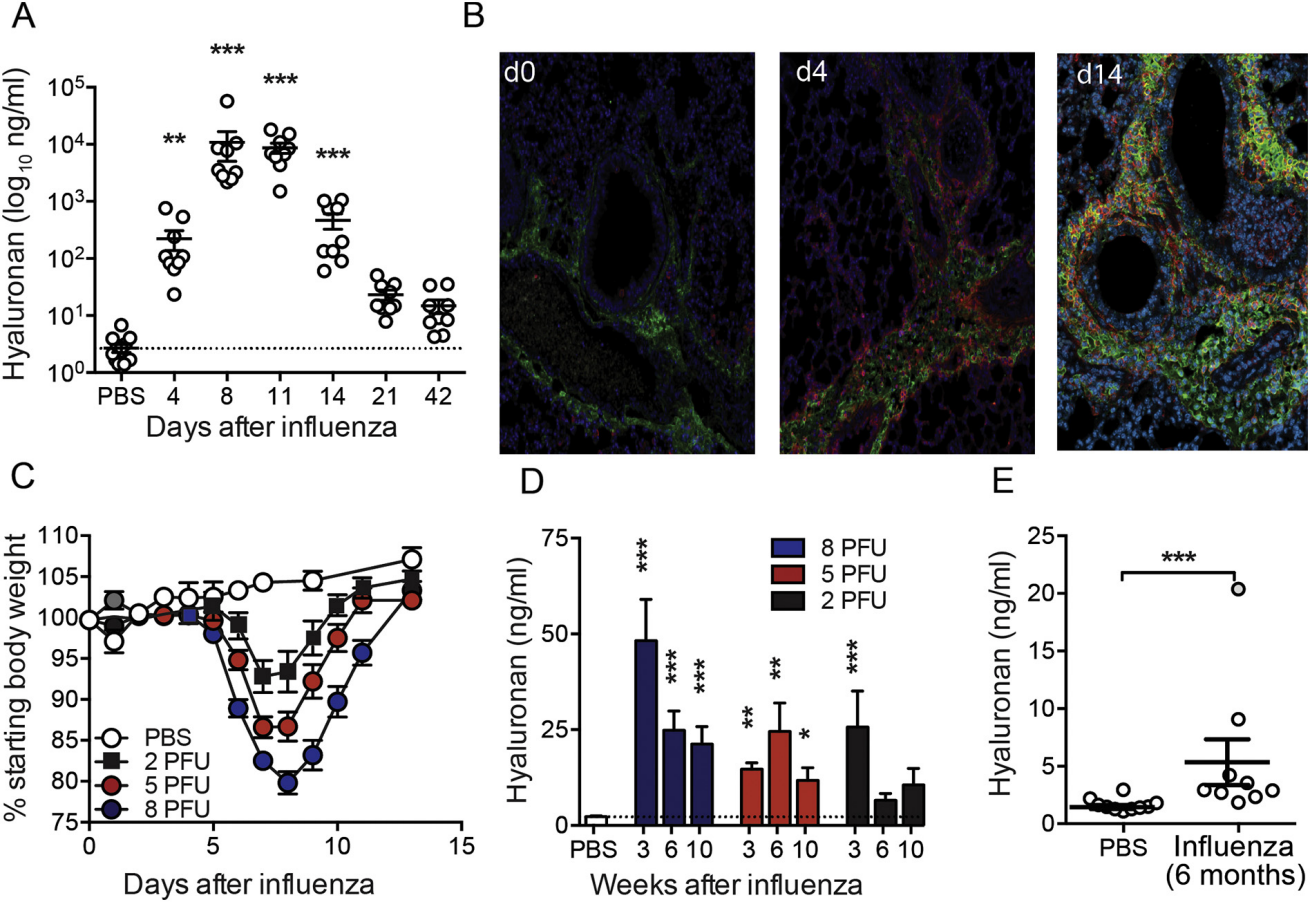
Hyaluronan accumulation in resolution of influenza infection. Mice were infected with 6 PFU PR8 influenza and airway hyaluronan content determined in BAL fluid by ELISA (A). Paraffin embedded formalin-fixed lung sections from mice infected with 6 PFU PR8 influenza were stained for hyaluronan using bHABP (green), CD44 (red) and nuclei using DAPI (blue) (B). Mice were infected with 2, 5 or 8 PFU PR8 influenza, weight monitored daily (C), and BAL hyaluronan content determined by ELISA after 3, 6 and 10 weeks (D). Hyaluronan content was also measured in BAL fluid from mice 6 months after treatment with PBS or infection with influenza (E). Error bars represent mean ± SEM. *n* = 9–13 mice/group (A), representative images from *n* = 3 mice (B), *n* = 12 mice/group (C), n = 9–17 mice/group (D) and n = 9–10 mice/group (E). * = *p* < 0.05, ** = *p* < 0.01, *** = *p* < 0.001 by ANOVA with Dunn's post-test (a), ANOVA with Holm-Sidak post-test (d) or Mann-Whitney U test (E).

During H1N1 Influenza A virus (PR8) infection of C57BL/6 mice, hyaluronan in airway lavage fluid increased approximately 80- and 4000-fold on days 4 and 8, respectively (Fig. 1A) and remained elevated above PBS controls 21 and 42 days later. Influenza infection was also associated with a redistribution of HA from a thin distinct sub-epithelial location in uninfected mice to excess deposition including the airways during influenza infection (Fig. 1B). Histological analysis also revealed that luminal and interstitial hyaluronan contained numerous CD44-expressing airway macrophages (Fig. 1B).

We next assessed whether hyaluronan accumulation was dependent on the severity of disease or the type of airway infection. 2, 5 or 8 plaque forming units (pfu) of influenza A PR8 infection produced weight loss of different severity as expected (Fig. 1c). In all cases accumulation of hyaluronan was observed in airway lavage fluid that persisted at 10 weeks after infection (Fig. 1d). Whilst not statistically significant after correcting for multiple comparisons, hyaluronan levels were still 3-fold higher than that of naïve mice 10 weeks after infection with the lowest dose of 2 PFU influenza virus (Fig. 1d). Remarkably, airway hyaluronan content was also still significantly elevated 6 months after 6 PFU influenza virus infection (Fig. 1e) demonstrating long-term effects on lung hyaluronan homeostasis. This prolonged elevation in airway hyaluronan was also observed following RSV infection, in an eosinophilic and neutrophilic model of airway *Cryptococcus neoformans* infection and in samples from stable and exacerbating COPD patients confirmed as having influenza infection (Fig. 1E).

### Hyaluronan metabolism is altered after influenza infection

**Fig. 2:**
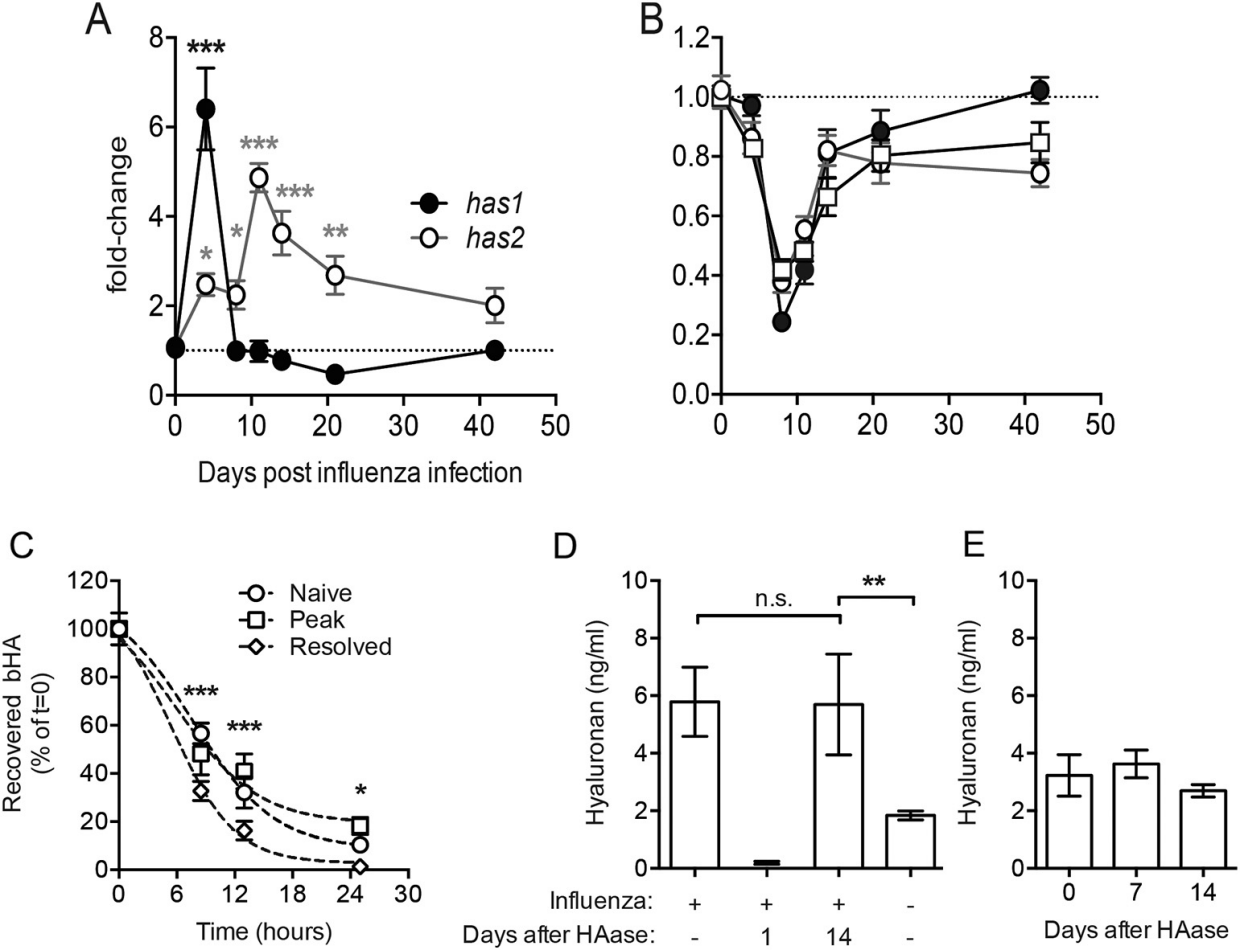
Accumulation of hyaluronan after influenza infection is due to excess expression of hyaluronan synthases and not due to defects in clearance. Expression of hyaluronan synthases 1 & 2 (a; *has1* & *has2*) and hyaluronidases 1–3 (b; squares, open circles and closed circles, respectively) was determined in whole-lung RNA extracts. Recovery of bHA in BAL fluid after intranasal administration with 20 μg bHA in naïve mice, mice at the peak of influenza infection (day 8) and influenza-resolved mice (day 14 after infection) was measured by ELISA (C). Mice were treated with *Streptomyces* hyaluronidase three weeks after influenza infection, and BAL hyaluronan measured after 1–14 days in treated mice, in untreated infected mice and in naïve untreated mice (D). BAL hyaluronan was also measured 7 and 14 days after hyaluronidase treatment of naïve mice (E). Relative gene expression is shown as fold-change relative to naïve (day 0) control groups after normalisation to the control genes *hprt* and *gapdh*. Error bars represent mean ± SEM. *n* = 8–12 mice/group (a,b), *n* = 3–5 mice/group (c) and *n* = 7–9 mice/group (D,E). * = *p* < 0.05, ** = *p* < 0.01, *** = *p* < 0.001 vs control group by ANOVA with Holm-Sidak post-test (A,B). * = *p* < 0.05, *** = *p* < 0.001 between naïve and resolved mice, and # = *p* < 0.05 between naïve and peak-infected mice by 2-way ANOVA with Dunnet's post-test (c). * = *p* < 0.05, ** = *p* < 0.01 by ANOVA with Dunn's post-test (D).

Accumulation of hyaluronan during infectious lung disease may represent excess synthesis and/or reduced degradation. We therefore next determined the expression of hyaluronan synthase (*has1* & *has2*) and hyaluronidase (*hyal1*, *hyal2* & *hyal3*) genes by qPCR of whole lung RNA. Expression of *has1* peaked early during influenza infection at day 4 whereas *has2* expression peaked later and was maintained above naïve mice for at least 3 weeks (Fig. 2A). Concurrent to the increased expression of hyaluronan synthases, the expression of the three hyaluronidase genes examined was reduced by between 60 and 80% 8 days after infection, with *hyal1* & *hyal2* expression remaining suppressed for 6 weeks (Fig. 2B). Thus increased airway hyaluronan may represent enhanced synthesis and/or reduced degradation after resolution of influenza infection.

To assess the turnover of HA at different stages of infection, biotinylated hyaluronan (bHA) was administered to mice on day 8 or day 14 of influenza infection and the clearance of bHA from the airways over 24 h compared to uninfected mice. The half-life of bHA clearance was similar between naïve mice and those at day 8 of influenza infection (9.6 and 9.2 h, respectively) (Fig. 2C). However, bHA was cleared quicker in mice 14 days after influenza infection with only 1.4% of bHA remaining in the airways at 24 h, compared to 10.4% in naïve mice and 18.2% 8 days after influenza infection (Fig. 2C). Therefore hyaluronan clearance was not defective following influenza virus infection.

We next ascertained whether heightened production of hyaluronan occurred due to excess synthesis. To assess this we first cleared hyaluronan from the airways of mice 3 weeks after influenza infection by administering hyaluronidase once and then followed hyaluronan levels over 14 days. One day after hyaluronidase treatment airway hyaluronan was below even homeostatic levels observed in naïve mice. However, 14 days after hyaluronidase treatment, the amount of hyaluronan present had rebounded to a level indistinguishable from day 14 influenza-infected mice that had not been treated with hyaluronidase (Fig. 2D). This rebound was not due to hyaluronidase treatment per se as treatment had no effects on airway hyaluronan in naïve mice (Fig. 2E). This clearly demonstrates that post-influenza airway hyaluronan levels can be manipulated, but not ‘reset’ and that hyaluronan levels are maintained by excess production in the lung.

### Which cells are responsible for HA synthase production?

**Fig. 3:**
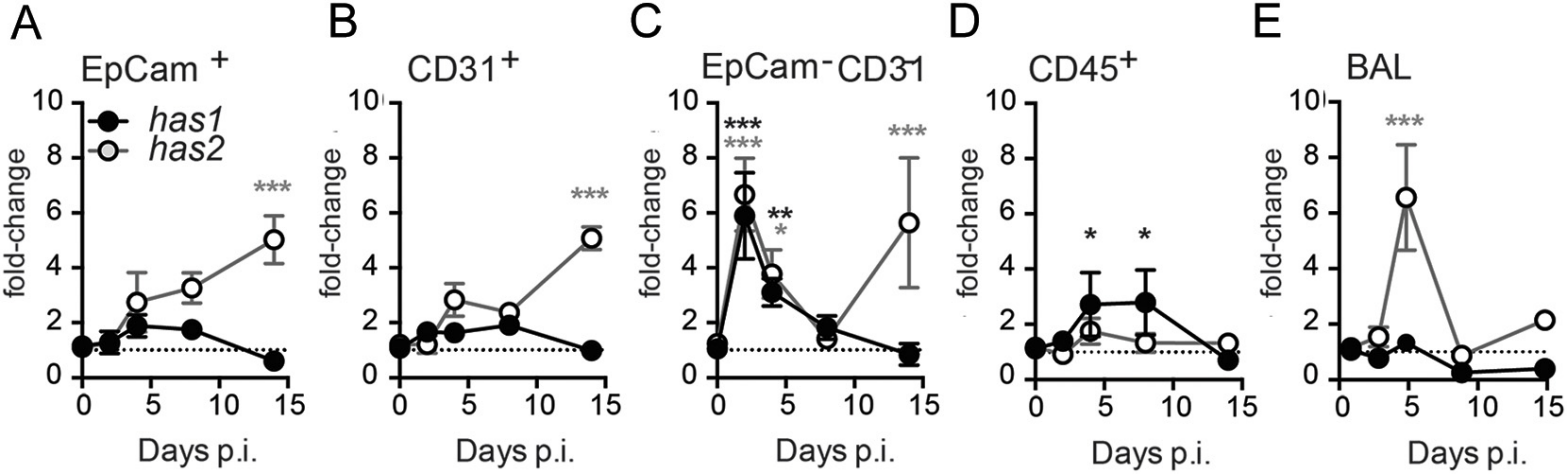
Hyaluronan synthase and hyaluronidase expression during influenza infection. Expression of hyaluronan synthases 1 & 2 (*has1* & *has2*) was determined in sorted lung populations (A-D) and whole-BAL (E) RNA extracts by qPCR. Relative gene expression is shown as fold-change relative to naïve (day 0) control groups after normalisation to the control genes *hprt* and *gapdh*. Error bars represent mean ± SEM. *n* = 6–7 (A-D) and n = 8–12 (E) mice/group. * = *p* < 0.05, ** = *p* < 0.01, *** = *p* < 0.001 vs control group by ANOVA with Holm-Sidak post-test.

The studies above suggest that to tackle accumulated hyaluronan following influenza infection its production would need to be targeted. We therefore examined cells expressing hyaluronan synthases and hyaluronidases by sorting CD45^−^EpCam^+^ epithelial cells, CD45^−^CD31^+^ endothelial cells, CD45^−^EpCam^−^CD31^−^ ‘stromal’ cells such as fibroblasts, and CD45^+^ ‘immune’ cells (Fig. 2) from homogenised lung tissue after influenza infection. As observed in Fig. 2a, the HA synthases 1 and 2 appeared with different kinetics: *has1* transiently at early time points and *has2* later in influenza infection resolution. EpCam^−^CD31^−^ stromal cells and CD45^+^ immune cells were the predominant source of *has1*, whereas *has2* was observed in EpCam^−^CD31^−^ stromal, CD31^+^ endothelial and EpCam^+^ epithelial cells (Fig. 3a-d). In airway lavage (BAL), *has1* mRNA was below the threshold of detection, whereas *has2* followed a similar kinetics to that observed in lung homogenate EpCam^−^CD31^−^ stromal cells at early time points (Fig. 3e). Long term alterations of hyaluronan following influenza are therefore likely due to prolonged HA synthesis by non-immune structural cells in the lung.

### Influenza infection induces the formation of hyaluronan-protein complexes

**Fig. 4:**
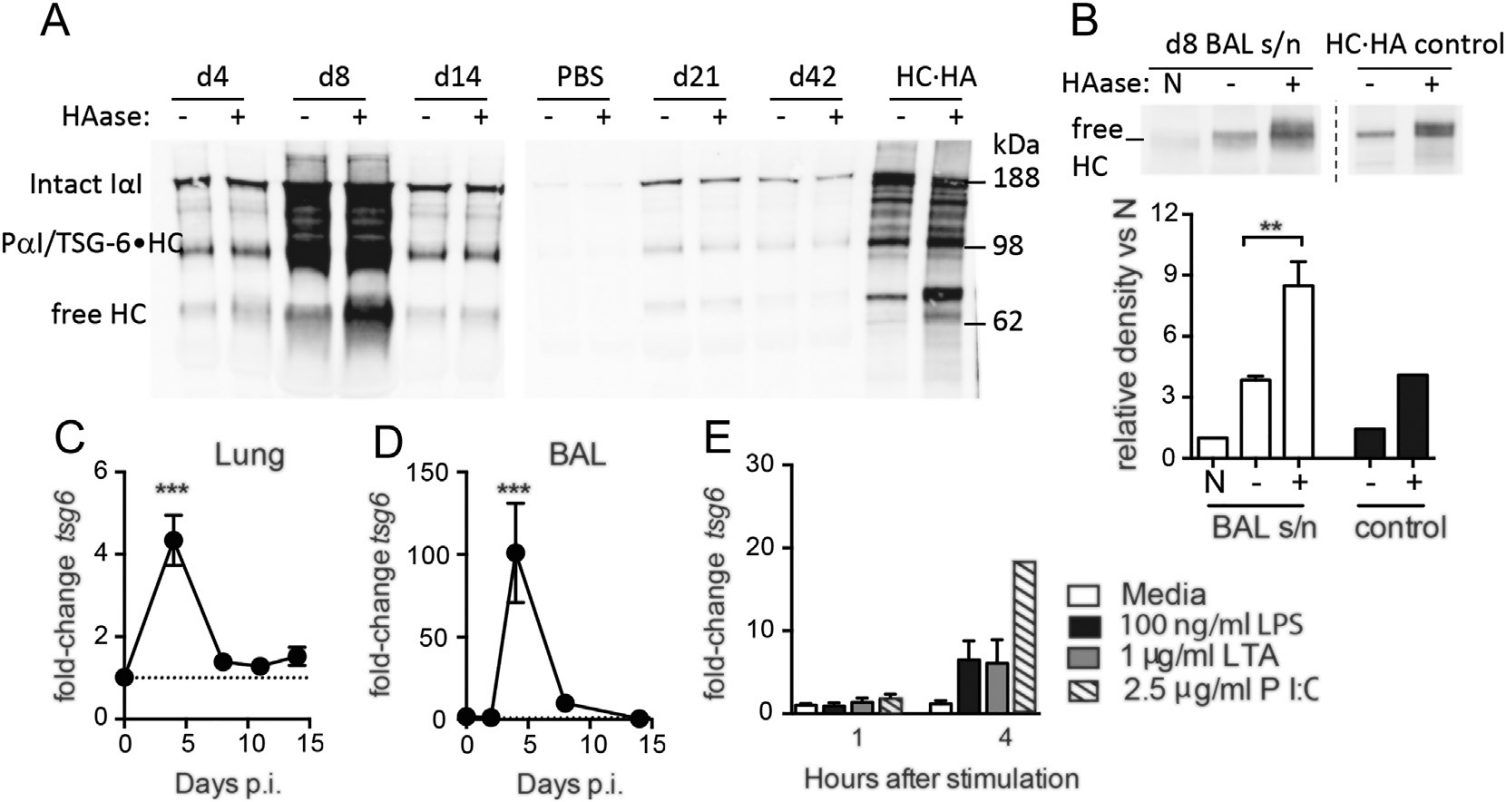
Heavy chain-hyaluronan complexes are generated after viral infection. BAL supernatants from influenza infected mice were incubated at 60 °C for 30 min in the presence (+) or absence (−) of *Streptomyces* hyaluronidase to release free HCs from HC·HA complexes, and intact IαI and free HCs detected by western blot (A). Additional BAL samples from day 8 after infection were either incubated on ice (N) or incubated for 30 min at 60 °C in the absence (−) or presence (+) of hyaluronidase, then free HCs detected by western blot and relative band densities quantified using ImageJ (B). HC·HA synthesised in vitro [94] was also hyaluronidase treated in parallel as a control (A,B). The expression of *tsg6* mRNA was determined in whole-lung (C) and whole-BAL (D) RNA extracts from influenza-infected mice, and in isolated alveolar macrophages stimulated *ex vivo* with LPS, LTA or Poly(IC) (E), by qPCR. Representative blots shown from *n* = 2 (A) or *n* = 5 (B) mice, with boundaries between individual blots highlighted. Error bars represent mean ± SEM. n = 5 (B), *n* = 9–12 (C), *n* = 6–7 (D) and n = 3–5 (E). * = *p* < 0.05, ** = *p* < 0.01, *** = *p* < 0.001 by Mann-Whitney *U* test (B) or ANOVA with Holm-Sidak post-test (C-E).

During airway inflammation hyaluronan is often covalently modified by the addition of HC from IαI via the enzyme TSG-6 [3] leading to a “pathological matrix” that promotes cellular adhesion and myofibroblast differentiation [55, 56]. This occurs via formation of a covalent TSG-6·HA intermediate [57]. Surprisingly, the presence of this modification has not been examined before during lung viral infection *in vivo*. The low amounts of hyaluronan present in naive mouse airways precluded further analysis. However, bands corresponding to intact IαI and a smaller IαI-immunoreactive species (likely corresponding to pre-α-inhibitor (PαI) and/or TSG-6·HC) were strongly enriched after infection (Fig. 4A). Hyaluronidase digestion of lung and airway lavage samples prior to western blot significantly enriched a band corresponding to free HCs at day 8 after infection (Fig. 4A,B, respectively). As hyaluronan and hyaluronan-protein complexes are too large to run into a polyacrylamide gel, the enrichment of a free HC band after hyaluronidase digestion strongly suggests that HC·HA complexes are present in the lung tissue and BAL fluid at day 8 after infection.

TSG-6 transfers heavy chains from IαI to hyaluronan and the *tsg6* gene was significantly elevated ~4-fold in whole-lung RNA isolates (Fig. 4C) and 100-fold in whole-BAL RNA isolates (Fig. 4D) at day 4 after infection. As we observed a strong increase in *tsg6* expression in BAL cells of influenza-infected mice, we investigated whether alveolar macrophages were capable of expressing *tsg6* in response to inflammatory stimuli. Treatment of isolated alveolar macrophages with the viral TLR3 mimetic Poly(IC), and to a lesser extent the bacterial TLR agonists lipopolysaccharide (LPS) and lipoteichoic acid (LTA), resulted in the strong expression of *tsg6* (Fig. 4E). Therefore, not only does HA persist after influenza virus infection, but it is present in a modified form.

**Fig. 5:**
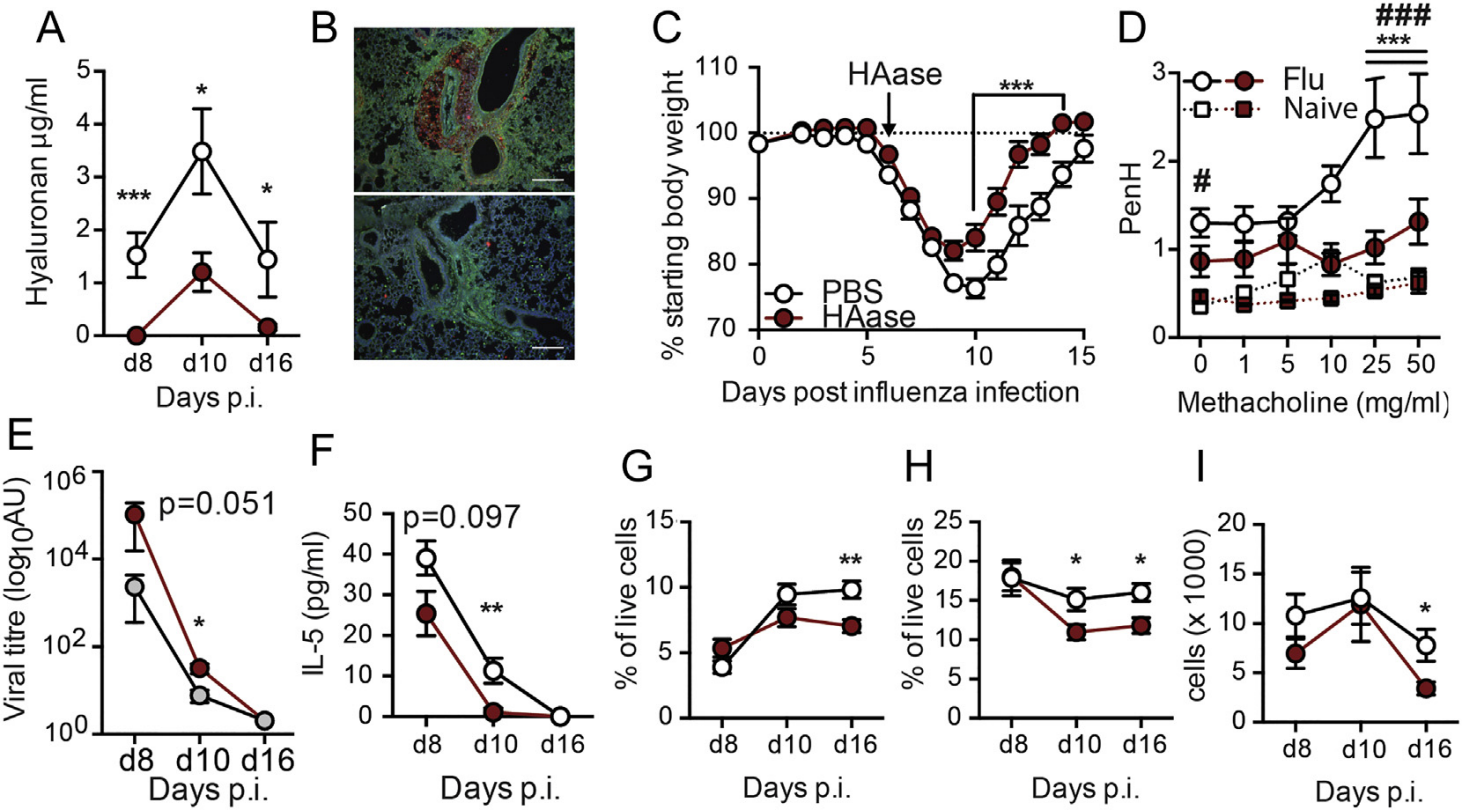
Reducing hyaluronan accumulation improves recovery after influenza infection. Mice were infected with influenza, and then given a single dose of PBS or hyaluronidase intranasally on day 6. BAL fluid hyaluronan content was determined in heat inactivated BAL samples by ELISA (A). Representative lung sections from influenza infected mice treated with PBS (top) or hyaluronidase (bottom) were stained for DAPI (blue), CD44 (Green) and HA (red). Scale bar represents 500 μm (B). Body weight was monitored daily and is represented as percent original weight (C). Lung function was measured by whole body plethysmography under increasing doses of methacholine at day 16 after infection (D). Viral titres in whole-lung RNA extracts were measured by qPCR for viral RNA (E). IL-5 was measured in BAL fluid by CBA (F). The proportion of CD4+ T cells (G), F4/80^+^CD11b^+^SiglecF-CD11c^low^ recruited macrophages (H) and number CD11c^−^SiglecF^+^ eosinophils (I) in the BAL was determined by flow cytometry. Error bars represent mean ± SEM. *n* = 7–20 mice/group (A,E-H), *n* = 20 mice/group (B), *n* = 4–6 mice/group (C), n = 6–10 (d). * = *p* < 0.05, ** = *p* < 0.01, *** = *p* < 0.001, comparisons PBS & HAase by 2-way ANOVA with Holm-Sidak post-test (B) or Mann-Whitney U test (A,D-H). *** = *p* < 0.001 between PBS and hyaluronidase treated influenza-infected mice and # = *p* < 0.05, ### = *p* < 0.001 between PBS treated naïve and influenza-infected mice by 2-way ANOVA with Holm-Sidak post-test (C).

### Reducing hyaluronan accumulation during influenza infection improves disease outcomes

Persistence of HA during infection may contribute to influenza virus-induced pathogenesis. We therefore next treated mice *in vivo* with a single dose of bacterial hyaluronidase prior to maximal weight loss on day 6 after infection. We were careful to ensure that our hyaluronidases were free of LPS contamination by purchasing from sources that proved purity by Mass Spectrometry, by confirming a lack of endotoxin ourselves by LAL assay and proving that they did not cause neutrophil recruitment when given intranasally (data not shown). Administration of hyaluronidase resulted in a significant reduction of HA at all subsequent time points tested **(**Fig. 5a**)**. Immune fluorescence analysis showed the extent of hyaluronan depletion in hyaluronidase treated mice, but little gross effect on cellularity **(**Fig. 5b**)** and accelerated weight recovery from day 9 compared to PBS treated influenza infected controls (Fig. 5c). Whole-body plethysmography at day 16 after infection showed that influenza virus infection in PBS treated control mice resulted in a worse lung function (i.e a significantly increased PenH baseline (0 mg/ml methacholine)), and a further PenH increase compared to naïve PBS treated mice given 25 and 50 mg/ml methacholine. Strikingly, hyaluronidase treatment reversed the effect of influenza on lung function (Fig. 5d). The reduction in weight loss and improved lung function observed after hyaluronidase treatment was not due to reduction in viral load, as hyaluronidase treated mice actually exhibited slightly impaired viral clearance 8–10 days after infection (Fig. 5e). Furthermore hyaluronidase treatment did not increase any negative regulators such as A20, IRAKM or Tollip in macrophages (data not shown). Nor were there any significant differences in TNF, MCP-1, IP-10, IL-6, IL-10, KC, IFN-γ, MIP-1α, MIP-1β, MIG or RANTES at any time after hyaluronidase treatment (Fig. 3), and we were unable to detect IL-1β, IL-4, IL-12p70, IL-13, IL-17A, IL-33, Nitric Oxide and TSLP. A small reduction in IL-5 was observed (Fig. 5f), but the functional outcome of this small decrease is unknown. A reduction of lung and airway cellularity can reduce the amount of weight loss during viral infection, but despite a partial reduction in CD4+ T cells (Fig. 5g), F4/80^+^CD11b^+^ macrophages (Fig. 5h) and CD11c^−^SiglecF^+^ cells (Fig. 5i), no differences were observed in any other cell type analysed (Fig. 4). Thus, hyaluronidase is beneficial during influenza infection and improves lung function without affecting viral induced inflammation.

## Discussion

This study demonstrates that following influenza virus infection hyaluronan production is elevated due to persistent HA synthase activity and that this contributes to prolonged impairment of lung function. Endogenous hyaluronidase is clearly not sufficient to cope with this excess production. Administration of additional exogenous hyaluronidase however restores lung function. Upon cessation of hyaluronidase treatment, HA levels return to their elevated state. Hyaluronidase is therefore a treatment, but does not rectify the underlying defect. It is possible that hyaluronidase treatment itself reduces HA to low molecular weight forms that stimulate further HA production. However, administration of high molecular weight HA with hyaluronidase did not result in excess HA production 7 or 14 days later (data not shown). Furthermore, the persistence of HA for weeks after clearance of influenza virus is associated with reduced hyaluronidase *in vivo*.

The level of hyaluronan produced titrated with the viral dose implying that long term impairment of lung function may only occur during a high dose infection, or in those unable to limit viral replication. Patients with underlying lung disease, including asthma and COPD, are more prone to severe respiratory viral infections [58, 59] that exacerbate their disease. Such exacerbations cause a worsening of symptoms, including a further reduction in lung function that requires additional treatment or even hospitalisation [60]. Respiratory fluids from patients with COPD contain higher levels of hyaluronan than healthy controls [61]. We now show that a concomitant viral infection in COPD patients and in mice exacerbates hyaluronan further, which may contribute to a worsening of lung function.

This raises the question of how excess hyaluronan would cause these effects. Though many studies examine the impact of hyaluronan molecular weight on inflammation [6], we did not observe any major cellular changes in hyaluronidase treated mice, except for small reductions in airway CD4^+^ T cells and interstitial macrophages. Rather, the improved lung function likely reflects a reduction in airway lumen hyaluronan that otherwise reduces airway volume. A similar improvement in airway hyper-reactivity is also observed in ozone-induced airways disease in CD44 or IαI deficient mice [62]. The ability of matrix to regulate cell positioning, retention and activation is well studied. We observed that influenza-induced airway hyaluronan deposition was associated with CD44-expressing macrophages that would also contribute to airway occlusion. Whether these macrophages are sequestered by excess HA or recruited with increased kinetics to it (as has been shown after macrophage stimulation with surfactant protein A [63] is unclear. In line with the sequestering theory, activated T cells induce hyaluronan production by human fibroblasts that results in cable-like structures of matrix that trap monocytes [64]. IL-1β also induces a hyaluronan rich extracellular matrix from fibroblasts that promotes monocyte binding [65]. Alternatively, enhanced macrophage numbers may reflect enhanced survival that is CD44-dependent [66]. These studies, together with others showing that hyaluronan and TSG-6 affect immune cell accumulation during lung inflammation [6, 14, 25, 41, 44, 64, [67], [68], [69], [70]], suggest that specific manipulation of hyaluronan or TSG-6 may provide a novel method to improve lung function in a wide variety of respiratory disorders.

Tracking the removal of administered biotinylated hyaluronan from the influenza-infected lung revealed that this process remained optimal. The accumulation of hyaluronan that we observed is more likely due to the prolonged expression of HA-synthases, particularly HAS2. Unfortunately, it is not possible to test *has2* knockout mice as they die at mid-gestation (E9.5-10) [71]. Whilst EpCam^+^ epithelial, CD45^−^CD31^−^EpCam^−^ stromal cells and CD31^+^ endothelial cells showed heightened *has2* expression in the resolution phase of influenza virus infection, *has1* mRNA peaked early in CD45^+^ and CD45^−^CD31^−^EpCam^−^ cells, but was not maintained. This differential profile of HA-synthase expression is consistent with that reported in mice challenged intranasally with ovalbumin (though the cellular source was not defined) [12] and agrees with the concept that expression of HA-synthases depends on the cell type [72, 73]. Transfection studies show that HA synthase isoforms differ in the duration of their activity. HAS1 is the most transiently expressed [74], with evidence suggesting that its expression and activity is induced by inflammation [75]. Furthermore a recent study suggests that maintenance of HAS2 expression prevents fibroblast senescence leading to enhanced fibrotic lung disease [76]. Such selective expression and kinetics of different HA synthase isoforms may allow selective manipulation to reduce continued matrix production whilst leaving other pathways intact.

The molecular mechanisms responsible for differential HA synthase isoform expression in our study is unclear, with the majority of the published literature using *in vitro* cell line assays. HAS2 expression is increased in an animal model of pulmonary hypertension [77], but decreased in pulmonary arterial smooth muscle cells in idiopathic pulmonary arterial hypertension where HAS1 is responsible for enhanced hyaluronan [78]. HA synthases can also be induced by endoplasmic reticulum stress [79]. TGF-β and TNF upregulate *has1* expression and activity in fibroblast-like synoviocytes [80], whilst IL-1β and TNF increase *has2* and hyaluronan production in lung fibroblasts [81]. Additionally, IFN-γ and TNF induce hyaluronan production in lung fibroblasts [82]. Since increased HA production is observed to a wide range of stimuli and in lung pathological conditions with very different aetiology and clinical characteristics (for a review see [21]), specificity may exist in which HA-synthase isoform(s) are induced. The specific inflammatory microenvironment, the type of antigen involved and the cells affected during disease may drive selective HA synthase expression. Our data also shows that the stage of inflammatory disease affects which HA-synthase dominates.

The amount of HA that accumulated correlated with disease severity in our influenza infection model suggesting at least a partial role in disease pathogenesis. We were surprised to find that only a single hyaluronidase treatment on day 6 of influenza virus infection was required to mediate faster reversal of weight loss and abrogation of bronchoconstriction to methacholine. An improvement in lung function may also reflect the liberation of IαI heavy chains from hyaluronan as we observed during hyaluronidase treatment of HC·HA complexes. IαI was barely detectable in PBS control mice, presumably because it is synthesised in the liver and does not traverse the healthy endothelial and epithelial barriers. However, IαI was enriched in the airways after influenza infection, and still raised six weeks later. TSG-6 facilitates IαI heavy chain transfer to HA [37] and it is interesting to note that TSG-6^−/−^ mice are resistant to the induction of airway hyper-responsiveness and show improved lung mechanics in response to methacholine challenge. TSG-6 is also implicated in hyaluronan deposition and airway hyper-responsiveness in allergic pulmonary inflammation [54]. In contrast, TSG-6 converts macrophages to an anti-inflammatory phenotype during LPS-induced lung injury by suppressing TLR4/NF-κB signalling [68], and it is reported as a potent anti-inflammatory protein. TSG-6 also inhibits neutrophil recruitment in response to inflammation [[83], [84], [85], [86]] and reduces nuclear NF-κB translocation in response to TLR-2 activation in a CD44- (and likely HA-) dependent manner [87].

With regards to excess and altered hyaluronan in the airspaces, there are multiple points that could be manipulated to reduce its role in pathology. These include, a reduction in its synthesis by HA synthase 2, the disruption of HC·HA complexes by hyaluronidase, inhibition of their formation or neutralisation of inflammatory mediators that up-regulate TSG-6. IαI heavy chains in the hyaluronan matrix can also be crosslinked by Pentraxin-3 [88] or express versican [89]; both are up-regulated in a variety of inflammatory lung diseases and amenable to manipulation. Recombinant human hyaluronidase is already licensed in the UK to enhance permeation of subcutaneous or intramuscular injections, local anaesthetics and subcutaneous infusions, to promote resorption of excess fluids and blood and to improve resorption of radiopaque agents in subcutaneous urography [90, 91]. To our knowledge, there are no studies examining the impact of hyaluronidase treatment on the long-term sequelae of acute inflammatory or chronic, lung conditions.

## Materials and methods

### Animals

Female BALB/C or C57BL/6 mice (Harlan, UK and Charles River, UK), 8–12 weeks of age, were kept in specific pathogen free conditions. To model viral infection, animals were anaesthetised with 2.5% isoflurane and infected intranasally with either 5–10 PFU of A/PR8/34 H1N1 influenza or 10^6^ plaque forming units (PFU) of RSV strain A in 50 μL sterile phosphate buffered saline (PBS). Animals were monitored daily and weight loss recorded.

### Plethysmography

Mice were placed in a whole-body plethysmograph (Buxco, UK) to facilitate the measurement of lung function as described previously [92]. Bronchoconstriction to aerosolised methacholine (Sigma-Aldrich, UK) at 1, 5, 10, 25 and 50 mg/ml for 60 s with 5 min intervals was determined.

### Sample recovery and processing

Animals were euthanized with 3 mg intraperitoneal pentobarbitone then exsanguinated via the femoral artery. Blood, lung homogenate and BAL fluid were collected and prepared as previously described [93]. BAL fluid was collected from the lungs by inflating *in situ* six times with 1.3 ml HBSS supplemented with 5 mM EDTA via an intratracheal cannula, centrifuged at 300 ×*g* for 5 min and supernatant stored at −80 °C for analysis by ELISA. Cell pellets were resuspended in 400 μL R10F (RPMI-1640 supplemented with 10% foetal calf serum and 1% penicillin/streptomycin). Lung lobes were shredded using scissors and incubated at 37 °C with 0.13 mg/ml Liberase (Roche, UK) and 50 μg/ml DNase I (Roche, UK) in R10F for 30 min, and the reaction stopped with 5 mM EDTA. Single cell suspensions were obtained by passing digests through a 70 μm sieve (BD labware, USA), and centrifuged for 5 min at 300 ×*g*. Red blood cells were lysed in ACK buffer (0.15 M ammonium chloride, 1 M potassium hydrogen carbonate and 0.01 mM EDTA, pH 7.2) for 3 min before centrifuging for 5 min at 300 ×g and resuspending in 1 mL R10F. Viable cells were counted manually by haemocytometer and trypan blue exclusion.

### Flow cytometry

Single-cell suspensions of lung digest or BAL cells were transferred to 96-well microtitre plates. Cells were washed in PBS then incubated for 30 min at 4 °C with a near-IR dead cell marker in PBS (Life Technologies, UK). Fc receptors were blocked using an anti-CD16/32, extracellular markers were stained with antibodies diluted in PBA (PBS/0.1% NaN_3_/1% BSA) for 20 min at 4 °C, and cells fixed with 2% paraformaldehyde at room temperature for 10 min. Data were acquired on either a BD FACS Canto II or BD Fortessa flow cytometer (BD Bioscience, UK) using FACS Diva software (BD Biosciences, Belgium). FlowJo software (Tree Star, USA) was used for data analysis.

In some experiments a BD Influx cell sorter (BD Bioscience, USA) was used to isolate specific cellular populations for further analysis. This was performed by the flow cytometry core facility at the Manchester Collaborative Centre for Inflammation Research (MCCIR, UK). Cells were collected into ice-cold PBS, spun at 2000 ×*g* for 5 min, resuspended in RLT lysis buffer (Qiagen, UK) and RNA extracted immediately.

### Hyaluronidase

Hyaluronidase from *Streptomyces hyalurolyticus* (Merck-Millipore, UK) was reconstituted at 240 U/ml in sterile PBS supplemented with 0.2 M CaCl_2_. Reconstituted hyaluronidase was passed through two separate EndoTrap HD 1/1 endotoxin removal columns (HyGlos, Germany), then passed through a 0.2 μm filter and aliquots stored at −20 °C. PBS (0.2 M CaCl_2_) that had been treated in the same way was used as a control in all *in vivo* experiments. Hyaluronidase was tested for the presence of endotoxin by LAL assay (Pierce, USA), as per the manufacturer's instructions. We only used hyaluronidase sources that were 1) below the threshold of detection by the LAL assay (Pierce, USA) and 2) contained a single peak by the manufacturers mass spec analysis. Naïve, influenza infected and influenza resolved mice were treated with 12 U hyaluronidase by intranasal inhalation. For all experiments utilising *in vivo* hyaluronidase treatment an aliquot of BAL supernatant was boiled at 95 °C for 10 min to inactivate any residual hyaluronidase activity prior to quantification of hyaluronan content by ELISA.

### Preparation of biotinylated hyaluronan

To generate biotinylated hyaluronan, 35 mg of high MW hyaluronan (R&D Systems, UK) was reconstituted at 1 mg/ml in MES buffer (0.1 M, pH 5; Sigma, UK) for 24 h at 4 °C with agitation. Sulpho-NHS (Sigma, UK), biotin hydrazide (Sigma, UK) and EDAC (Sigma, UK) were added to a final concentration of 0.184 mg/ml, 1 mM and 30 μM, respectively, and the reaction incubated at 4 °C overnight with agitation. The reaction was stopped by the addition of 1 ml of 4 M guanidine-HCl (Sigma, UK), and the resulting biotinylated hyaluronan (bHA) dialysed against 4 × 400 ml volumes of deionised H_2_O using 10 kDa molecular weight cut-off Slide-a-Lyser dialysis cassettes (Pierce). The recovered final volume was ~40 ml, indicating a final concentration of ~0.83 mg/ml, which was confirmed by hyaluronan Quantikine ELISA (R&D Systems) carried out following manufacturer's instructions. Single-use aliquots were stored at −20 °C. This protocol has been described to yield approximately 1 biotin molecules per 93 disaccharides of hyaluronan (Frost and Stern, 1997). For *in vivo* use, mice were treated with 20 μg bHA diluted in sterile PBS by intranasal inhalation under isoflurane anaesthesia.

### Detection of biotinylated hyaluronan

The hyaluronan ELISA from R&D Systems was modified to enable specific detection of bHA, rather than total HA. Plates were coated as normal with the supplied HA-binding protein, but instead of using the hyaluronan binding protein detection reagent, streptavidin-HRP (R&D Systems; diluted 1:100) was used to directly detect bound bHA. For a standard curve, bHA generated as described above was used to generate a 3-fold dilution series starting at 500 ng/ml. The detection limit was below 0.23 ng/ml. This modified ELISA was used to detect bHA in murine biological samples including BAL supernatants, lung homogenates and serum. Lung homogenates were generated by placing snap-frozen lung lobes in 500 μL sterile PBS, pulsing for 2 min at 22,000 rpm in a TissueLyserII (Qiagen, UK) and removing debris by spinning for 5 min at 500 ×*g*. Homogenates from mice treated with PBS rather than bHA were used to correct for the background detection of endogenous biotin in the lung.

### Quantification of soluble mediators

Soluble mediators were detected in BAL and tissue culture supernatants by enzyme-linked immunosorbent assay (ELISA), following the manufactures' instructions. Cytometric bead arrays (CBA; BD Bioscience, USA) were carried out on some samples using an adapted method for low bead and sample volume. Briefly, 0.2 μl of each CBA bead was combined and used with 5 μl of sample or standard in a total volume of 10 μl, then incubated at room temperature for 2 h in a 96-well conical bottom plate. Plates were then washed and incubated with 5 μl PE detection reagent for 1 h at room temperature. After washing and resuspending in 100 μl wash buffer, beads were acquired using a BD FACS Verse flow cytometer (BD Bioscience, USA). In these experiments, 16 different chemokines and cytokines were detected simultaneously.

### RNA extraction

For whole-lung RNA extracts, tissue was snap-frozen in liquid nitrogen and stored at −80 °C. Frozen lung tissue was lysed in 800 μl Trizol reagent (Life Technologies, UK) using a Qiagen TissueLyserII (Qiagen, UK) at 22,000 rpm for 2 min. Lysates were cleared by centrifugation at 12,000 ×*g* for 10 min, phases separated with chloroform (Sigma, UK), RNA precipitated with isopropanol (VWR, UK) and RNA washed with 70% ethanol (VWR, UK) as per the manufacturer's instructions. Washed and dried RNA pellets were resuspended in 40 μl DNase/RNase free molecular biology quality water (Life Technologies, UK) and RNA concentration quantified by nanodrop, then stored at −80 °C.

Isolated or cultured cells were lysed using RLT buffer (Qiagen, UK), homogenised using QIAshredder columns (Qiagen, UK) and RNA extracted by Qiagen RNeasy mini kit (Qiagen, UK) as per the manufacturer's instructions. RNA was eluted in 30 μl DNase/RNase free molecular biology quality water (Life Technologies, UK) and stored at −80 °C.

### Analysis of gene expression

cDNA was generated using the Applied Biosystems high-capacity RNA-to-cDNA kit (Life Technologies, UK), as per the manufacturer's instructions, using a 20 μl reaction in 96-well plates. 200 ng of RNA per reaction was used for whole-lung RNA extracts, and 5 μl of RNA used from sorted and cultured cells. After incubating for 1 h at 37 °C, and then 5 min at 95 °C, cDNA was diluted 1:10 in DNase/RNase free water (Life Technologies, UK) and stored at −20 °C.

To quantify relative levels of gene expression, qPCR was carried out in 384-well format using the Applied Biosystems TaqMan Fast Universal PCR Mastermix (Life Technologies, UK) in a 10 μl reaction volume using 4 μl of 1:10 diluted cDNA as a template. TaqMan probes for specific target genes (Life Technologies, UK) were used at the manufacturer recommended dilution. Probes: *hprt* Mm01545399_m1, *gapdh* Mm99999915_g1, *has1* Mm03048195_m1, *has2* Mm00515089_m1, *has3* Mm00515092_m1, *hyal1* (*nat6*) Mm00480053_m1, *hyal2* Mm00477731_m1, *hyal3* Mm00662097_m1.

Reactions were plated in duplicate, and plates run on a QuantStudio 12 K Flex qPCR thermocycler (Life Technologies, UK). Data were analysed manually in Excel (Microsoft, USA) using either 2^−dCT^ method to compare expression of genes relative to *gapdh*, or using 2^-ddCT^ (normalised to the average of *gapdh* and *hprt*) compared to control groups.

### Western blot

To digest HC·HA complexes for Western blotting, 20 μl of BAL supernatants (or control HC·HA - see below) were treated with 1 Unit of hyaluronidase from *S. hyalurolyticus* in 2 μl sodium acetate at 60 °C for 30 min or sodium acetate alone and reactions stopped with an equal volume of loading buffer and boiling for 10 min. Control HC·HA was generated by combining 1 μg high MW hyaluronan with 2 μg recombinant TSG-6 and 8 μg recombinant IαI in a 25 μl reaction containing 126 mM NaCl, 20 mM HEPES and 2 mM MgCl_2_ for 2 h at 4 °C and the reaction stopped for 15 min with 3 μl 100 mM EDTA and 2 μl water [94]. 40 μl (or 5 μl in the case of the control HC·HA) of digested BAL samples were boiled for 10 min in loading buffer under reducing conditions and run on a NuPAGE Bis-Tris 2–12% gel (Life Technologies, UK) for 2 h at 150 V. SeeBlue Plus2 pre-stained ladder was loaded to provide molecular weight markers. Samples were then transferred to a nitrocellulose membrane for 1 h, 60 V, membranes washed in PBS 0.1% Tween-20 (Sigma UK) and blocked with 10% milk, 0.1% Tween 20 and 0.02% BSA for 1 h at RT.IαI heavy chains were detected with 1:20,000 rabbit anti-human/mouse IαI heavy chain antibody (DAKO, USA) as described on [44] overnight at 4 °C, washed × 5 and bound antibody visualised with 1:5000 goat anti-rabbit 800CW infrared secondary antibody (Li-Cor, USA) for 1 Hr at RT. After washing blots were imaged using a Li-Cor Odyssey infrared scanner. Images were converted to grey scale, inverted and band densities quantified in Image J (NIH, USA). Ban densities for hyaluronidase treated samples were normalised to the respective untreated control sample and expressed as a fold change in band density.

### Immunofluorescence

Slides with 4 μM paraffin sections of primary murine lung tissue were simultaneously dewaxed, rehydrated and antigen retrieved in BioOptica W-CAP TEC buffer, pH 8.0 (Leica, USA) for 1 h at 95 °C, and allowed to cool at room temperature for 30 min. Slides were washed in PBS, and sections circled using a PAP pen (Sigma, UK). Control sections were then digested with 10 U/ml *S. hyalurolyticus* hyaluronidase in PBS for 2 h at 37 °C prior to staining to demonstrate specificity for hyaluronan. After 2× PBS washes, free biotin was blocked using a Biotin/Avidin blocking kit (Vector Labs, UK), and then sections blocked in PBS with 1% bovine serum albumin (BSA) for 1 h at room temperature.

After blocking, tissue sections were incubated with 5 μg/ml biotinylated hyaluronan binding protein (bHABP; Merck, UK) and 5 μg/ml rat anti-mouse CD44 (eBioscience, UK) overnight at 4 °C. Hyaluronidase-digested control sections were incubated with 5 μg/ml bHABP and 5 μg/ml rat IgG2b K isotype control antibody (eBioscience, UK). After primary antibody incubation, slides were washed twice in PBS and incubated for 45 min at room temperature with streptavidin conjugated AlexaFluor-488 and goat anti-rat IgG AlexaFluor-594 secondary antibodies (Fig. 1) or streptavidin conjugated AlexaFluor-555 and goat anti-rat IgG AlexaFluor-488 secondary antibodies (Fig. 5) (all Life Technologies, UK).

Stained slides were washed twice in PBS, then washed once in water and dried in a laminar flow hood in the dark for 15 min. Dried slides were mounted with ProLong Gold antifade containing DAPI (Life Technologies, UK) and stored at 4 °C. Images were acquired in the University of Manchester bioimaging core facility using an Olympus BX51 upright microscope with a Coolsnap EZ camera (Photometrics, UK) and MetaVue software (Molecular Devices, UK). A 10x/0.30 UPlanFLN objective was used, and specific filter sets for DAPI, FITC and Texas Red used to isolate individual channels.

### Human samples

Sputum samples from patients with COPD were obtained from the AstraZeneca Mölndal biobank under a materials transfer agreement with the University of Manchester, and originated from a previously published study [95]. Hyaluronan was detected by ELISA (R&D Systems, UK) as per the manufacturer's instructions. For samples containing dithiothreitol (DTT), standards were also diluted in the equivalent final concentration of DTT to account for any interference in hyaluronan detection.

### Statistics

GraphPad Prism was used for all statistical calculations. For multiple dataset analysis ANOVA with Holm-Sidak or Dunn's correction was applied. To compare two datasets paired or unpaired *t*-test were applied. Data are presented as the mean ± standard error of the mean (SEM). *P* values < 0.05 were considered significant (*p < 0.05, **p < 0.01, ***p < 0.001).

### Study approval

All animal experiments were performed under the regulations of the Home Office Scientific.

Procedures Act (1986), approved by both the Home Office and the local ethics committee of the University of Manchester and Imperial College London.

The following are the supplementary data related to this article.

**Supplementary Fig. 1:**
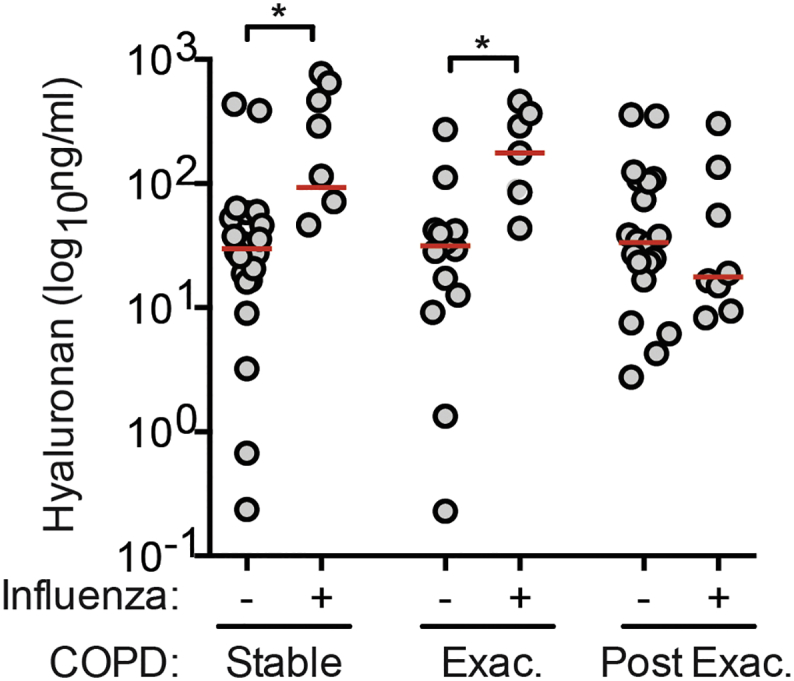
Hyaluronan is increased in sputum samples from influenza-positive COPD patients. Hyaluronan content in sputum from COPD patients was elevated in samples PCR-positive for influenza. Influenza status was determined by PCR, and samples stratified into three groups based on COPD disease state at time of sample collection: Stable (*n* = 29), Exacerbation (Exac.) (*n* = 20) and 2–6 weeks Post-Exacerbation (Post Exac.) (*n* = 28). Line indicates the median. * = *p* < 0.05 by Mann-Whitney *U* test. All Hyaluronan measurement was by ELISA.

**Supplementary Fig. 2:**
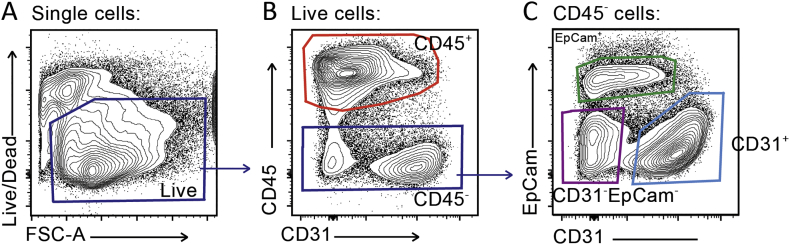
Gating plan for isolation of lung cell populations by flow cytometry. To sort immune, epithelial, endothelial and lineage negative stromal cells by flow cytometry, whole lung was digested with collagenase and DNase then homogenised to single cell suspension. Viable cells were selected (a), and CD45+ immune cells collected (b). Of the CD45- cells, EpCam+ epithelial cells, CD31+ endothelial cells and CD31-EpCam- stromal cells were collected (c). Representative plots shown.

**Supplementary Fig. 3:**
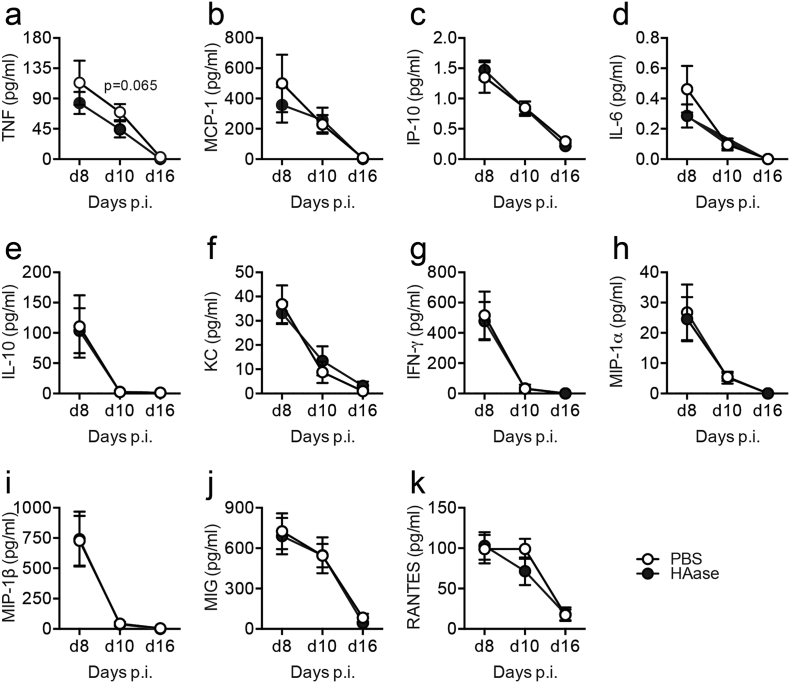
Cytokines in the airways after hyaluronidase treatment in influenza-infected mice. Mice were infected with influenza, then given a single dose of either PBS or hyaluronidase by intranasal inoculation on day 6. BAL fluid TNF (a), MCP-1 (b), IP-10 (c), IL-6 (d), IL-10 (e), KC (f), IFN-γ (g), MIP-1α (h), MIP-1β (i), MIG (j) or RANTES (k) content was determined in BAL samples by ELISA or CBA. Error bars represent mean ± SEM, *n* = 7–20 mice/group.

**Supplementary Fig. 4:**
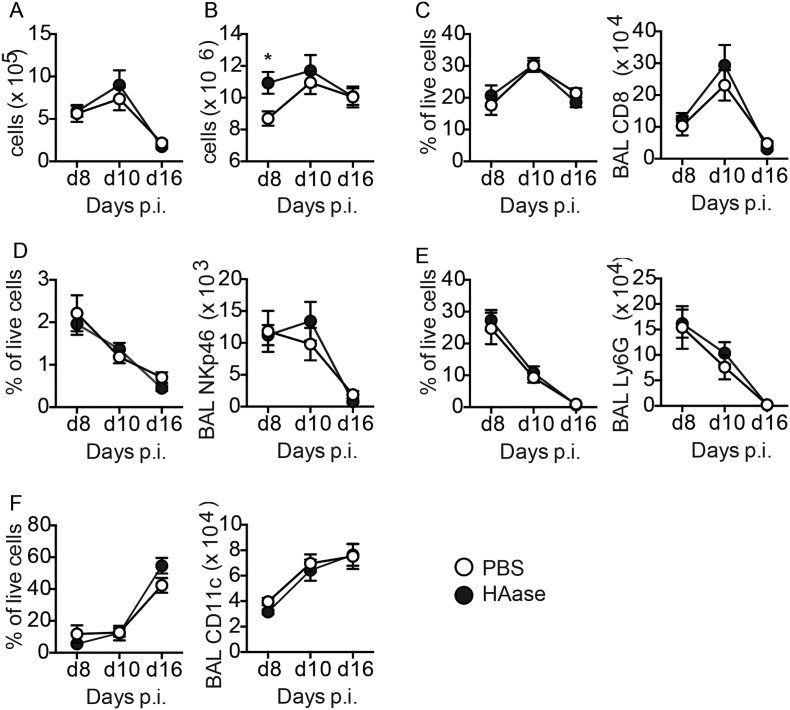
Cell numbers in the lung and airways after hyaluronidase treatment in influenza-infected mice. Mice were infected with influenza, then given a single dose of either PBS or hyaluronidase by intranasal inoculation on day 6. BAL cell number (a), lung cell number (b) and the number and proportion of CD8+ T cells (c), NKp46+ NK cells (d), F4/80-Ly6G+ neutrophils (e) and CD11c + SiglecF+ alveolar macrophages (f) was determined by flow cytometry. Error bars represent mean ± SEM, n = 7–20 mice/group. * = *p* < 0.05 by Mann-Whitney *U* test.
